# Bat Coronaviruses in China

**DOI:** 10.3390/v11030210

**Published:** 2019-03-02

**Authors:** Yi Fan, Kai Zhao, Zheng-Li Shi, Peng Zhou

**Affiliations:** 1 CAS Key Laboratory of Special Pathogens and Biosafety, Wuhan Institute of Virology, Chinese Academy of Sciences, Wuhan 430071, China; yifanfs0224@163.com (Y.F.); chiukal@163.com (K.Z.); zlshi@wh.iov.cn (Z.-L.S.); 2 University of Chinese Academy of Sciences, Beijing 100049, China

**Keywords:** coronavirus, bat, epidemiology, cross-species, zoonosis

## Abstract

During the past two decades, three zoonotic coronaviruses have been identified as the cause of large-scale disease outbreaks–Severe Acute Respiratory Syndrome (SARS), Middle East Respiratory Syndrome (MERS), and Swine Acute Diarrhea Syndrome (SADS). SARS and MERS emerged in 2003 and 2012, respectively, and caused a worldwide pandemic that claimed thousands of human lives, while SADS struck the swine industry in 2017. They have common characteristics, such as they are all highly pathogenic to humans or livestock, their agents originated from bats, and two of them originated in China. Thus, it is highly likely that future SARS- or MERS-like coronavirus outbreaks will originate from bats, and there is an increased probability that this will occur in China. Therefore, the investigation of bat coronaviruses becomes an urgent issue for the detection of early warning signs, which in turn minimizes the impact of such future outbreaks in China. The purpose of the review is to summarize the current knowledge on viral diversity, reservoir hosts, and the geographical distributions of bat coronaviruses in China, and eventually we aim to predict virus hotspots and their cross-species transmission potential.

## 1. Introduction

Fifteen years after the first highly pathogenic human coronavirus caused the severe acute respiratory syndrome coronavirus (SARS-CoV) outbreak, another severe acute diarrhea syndrome coronavirus (SADS-CoV) devastated livestock production by causing fatal diseases in pigs. Both outbreaks began in China and were caused by coronaviruses of bat origin [1,2]. This increased the urgency to study bat coronaviruses in China to understand their potential of causing another virus outbreak.

In this review, we collected information from past epidemiology studies on bat coronaviruses in China, including the virus species identified, their host species, and their geographical distributions. We also discuss the future prospects of bat coronaviruses cross-species transmission and spread in China.

## 2. Why Study Bat Coronaviruses in China?

### 2.1. Coronavirus Taxonomy

Coronaviruses (CoVs) belong to the subfamily Orthocoronavirinae in the family Coronaviridae and the order Nidovirales. CoVs have an enveloped, crown-like viral particle from which they were named after. The CoV genome is a positive-sense, single-strand RNA (+ssRNA), 27–32 kb in size, which is the second largest of all RNA virus genomes. Typically, two thirds of the genomic RNA encodes for two large overlapping polyproteins, ORF1a and ORF1b, that are processed into the viral polymerase (RdRp) and other nonstructural proteins involved in RNA synthesis or host response modulation. The other third of the genome encodes for four structural proteins (spike (S), envelope (E), membrane (M), and nucleocapsid (N)) and other accessory proteins. While the ORF1a/ORF1b and the four structural proteins are relatively consistent, the length of the CoV genome is largely dependent on the number and size of accessory proteins [3].

Compared with other RNA viruses, the expanded genome size of CoVs is believed to be associated with increased replication fidelity, after acquiring genes encoding RNA-processing enzymes [4]. Genome expansion further facilitates the acquisition of genes encoding accessory proteins that are beneficial for CoVs to adapt to a specific host [5]. As a result, genome changes caused by recombination, gene interchange, and gene insertion or deletion are common among CoVs. The CoV subfamily is expanding rapidly, due to the application of next generation sequencing which has increased the detection and identification of new CoV species. As a result, CoV taxonomy is constantly changing. According to the latest International Committee of Taxonomy of Viruses (ICTV) classification, there are four genera (α-, β-, δ-, and γ-) consisting of thirty-eight unique species in the subfamily [6]. The number of species will continue to increase, as there are still many unclassified CoVs [7,8].

CoVs cause disease in a variety of domestic and wild animals as well as in humans, where α- and β-CoVs mainly infect mammals and γ- and δ-CoVs mainly infect birds (Table 1). Two highly pathogenic β-CoVs, SARS-CoV, and MERS-CoV have caused pandemics in humans since 2002 [1,9]. Originating in China and then spreading to other parts of the world, SARS-CoV infected around 8000 individuals with an overall mortality of 10% during the 2002–2003 pandemic [1]. Since its emergence in 2012 in the Middle East, MERS-CoV spread to 27 countries, resulting in 2249 laboratory-confirmed cases of infection with an average mortality of 35.5% (until September 2018) [9]. Besides these two viruses, α-CoVs 229E and NL63 and β-CoVs OC43 and HKU1 can also cause respiratory diseases in humans [10]. Moreover, CoVs cause pandemic disease in domestic and wild animals (Table 1). SADS-CoV was recently identified as the etiological agent responsible for a large-scale outbreak of fatal disease in pigs in China that caused the death of more than 20,000 piglets [2]. Porcine epidemic diarrhea virus (PEDV) and transmissible gastroenteritis virus (TGEV) that belong to α-CoV and porcine δ-CoV (PDCoV) are also important emerging and re-emerging viruses in pigs that pose significant economic threat to the swine industry [11]. In addition, avian infectious bronchitis virus (IBV, γ-CoV) causes a highly contagious disease that affects poultry production worldwide [12]. Coronaviruses have also been associated with catarrhal gastroenteritis in mink (MCoV) and whale deaths (BWCoV-SW1) [13,14].

### 2.2. Linking Bats to Coronaviruses

Bat are the only mammals with the capability of powered flight, which enables them to have a longer range of migration compared to land mammals. Bats are also the second largest order of mammals, accounting for about a fifth of all mammalian species, and are distributed worldwide. Phylogenetic analysis classified bats into two large suborders—the Yinpterochiroptera, consisting of one Pteropodidae (megabat) and five Rhinolophoidea (microbat) families, and the Yangochiroptera comprising a total of thirteen microbat families [15].

It is hypothesized that flight provided the selection pressure for coexistence with viruses, while the migratory ability of bats has particular relevance in the context of disease transmission [16]. Indeed, bats were linked to a few highly pathogenic human diseases, supporting this hypothesis. Some of these well characterized bat viruses, including bat lyssaviruses (Rabies virus), henipaviruses (Nipah virus and Hendra virus), CoVs (SARS-CoV, MERS-CoV, and SADS-CoV), and filoviruses (Marburg virus, Ebola virus, and Mengla virus), pose a great threat to human health [16,17]. A comprehensive analysis of mammalian host–virus relationships demonstrated that bats harbor a significantly higher proportion of zoonotic viruses than other mammalian orders [18]. Viruses from most of the viral families can be found in bats [16].

Bats are now recognized as important reservoir hosts of CoVs (Table 1). Although civet cats were initially identified as the animal origin of SARS-CoV, bats were soon found to be the most likely natural reservoir hosts of this virus [19,20,21]. Long-term surveillance revealed an average 10% SARS-related CoV nucleotide positivity in bats, including some viruses that can use same human entry receptor ACE2 as SARS-CoV [7,22]. Similarly, bats have been proposed to harbor the progenitor viruses of MERS-CoV, although dromedary camels can transmit this virus to humans directly [9]. The most recent SADS-CoV spillover was traced back to bats [2]. In addition, bats also carry α-CoVs that are related to pathogenic human 229E- and NL63-CoVs, as well as pandemic swine coronavirus PEDV [23,24]. In summary, bats carry major α- (10 out of 17) and β- (7 out of 12) CoV species that may spillover to humans and cause disease (Table 1). Attributed to the wide distribution of bats, CoVs can be found worldwide, including China [25].

### 2.3. Why China?

Two bat CoVs caused outbreaks in China; it is thus urgent to study the reasons to avoid future outbreaks. China is the third largest territory and is also the most populous nation in the world. A vast homeland plus diverse climates bring about great biodiversity including that of bats and bat-borne viruses—most of the ICTV coronavirus species (22/38) were named by Chinese scientists studying local bats or other mammals. The majority of the CoVs can be found in China (Table 1). Moreover, most of the bat hosts of these CoVs live near humans, potentially transmitting viruses to humans and livestock. Chinese food culture maintains that live slaughtered animals are more nutritious, and this belief may enhance viral transmission.

It is generally believed that bat-borne CoVs will re-emerge to cause the next disease outbreak. In this regard, China is a likely hotspot. The challenge is to predict when and where, so that we can try our best to prevent such outbreaks.

## 3. Bat Coronaviruses That Are Associated with Diseases

### 3.1. SARS-Related Coronaviruses

**Figure 1:**
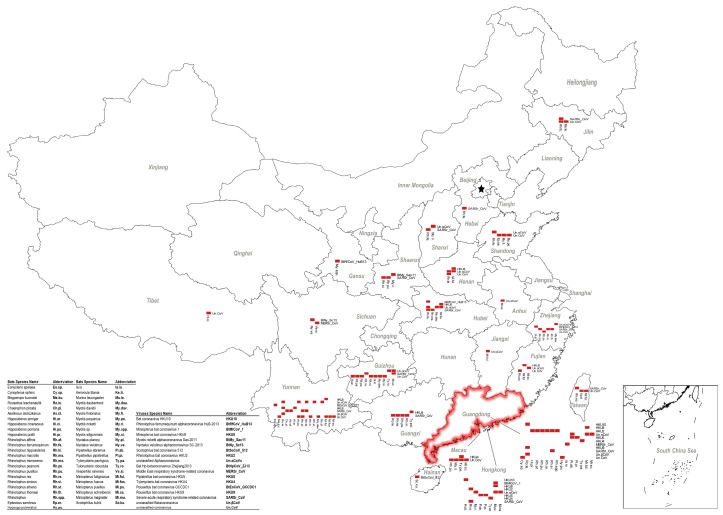
Geographical distribution of bat coronaviruses (CoVs) and their corresponding bat hosts in China. Each red box represents one CoV positive sample found in that particular bat species. One dot matrix was drawn for each province where a CoV positive sample had been reported. Guangdong Province, where SARS and SADS began, is circled in red. Abbreviations of bat species and virus species are indicated.

In November 2012, the first case of SARS was recorded in Foshan city, Guangdong Province, China (Figure 1). In 2005, two independent Chinese groups reported the first bat SARS-related CoV (SARSr-CoV) that was closely related to human SARS-CoV, implying a bat origin of the latter [20,21]. Since then, more bat SARSr-CoV isolates were identified in China (Table 1). Genome identities of these bat SARSr-CoVs are as high as 92% to human SARS-CoV, but their major receptor binding spike proteins cannot use the human virus entry receptor ACE2 [67]. Whether they are the progenitor viruses of SARS-CoV is debatable. In 2013, the isolation of a bat SARSr-CoV that uses the ACE2 receptor provided the strongest evidence of the bat origin of SARS-CoV [22]. Furthermore, the building blocks for SARS-CoV were identified from eleven different SARSr-CoV viral strains in a five-year surveillance program in a cave inhabited by multiple species of horseshoe bats in Yunnan Province, China [62].

**Figure 2:**
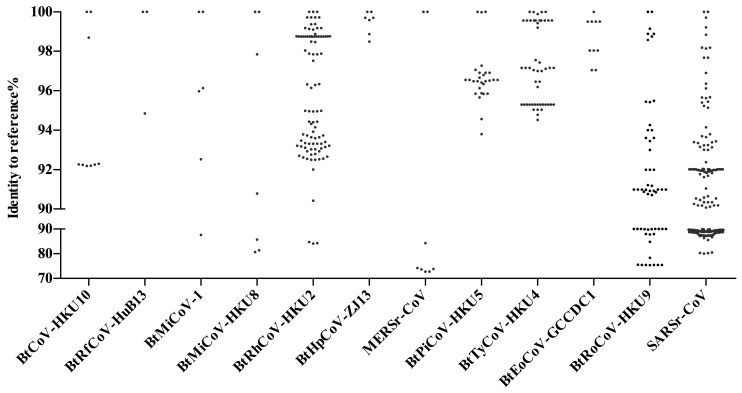
Genetic diversity of bat CoV in China. Sequences of 440 bp conserved the viral polymerase (RdRp) region for each CoV species were compared to related reference sequences. Reference genomes used: BtCoV-HKU10, NC_018871.1; BtRfCoV-HuB13, NC_028814.1; BtMiCoV-1, EU420138.1; BtMiCoV-HKU8, NC_010438.1; BtRhCoV-HKU2, MF094682.1; BtHpCoV-ZJ13, NC_025217.1; MERSr-CoV, NC_038294.1; BtPiCoV-HKU5, NC_009020.1; BtTyCoV-HKU4, NC_009019.1; BtRoCoV-GCCDC1, MG762606.1; BtRoCoV-HKU9, NC_009021.1; and SARSr-CoV, NC_004718.3. Notably, samples that were positive for BtMy-Sax11, BtNy-Sc13, and BtScCoV-512 were also identified in China. These were not taken into account here as too few sequences were available.

SARSr-CoVs found in China show great genomic diversity (Figure 2). Sequence identities of the conserved 440 bp RdRp region ranges from 80 to 100% with human SARS-CoV. CoV diversity in bats is thought to be shaped by both species richness and geographical distribution, and CoVs exhibit clustering at the bat genera level, with these genus-specific clusters largely associated with distinct CoV species [25]. Our analysis supports this theory. SARSr-CoVs are present in different bat species but all belong to the family of Rhinolophidae and Hipposideridae (Figure 1). *Chaerephon plicata* bats were also reported as carriers in one study, but this cannot be conclusively supported without molecular identification of the bat species [8]. In China, horseshoe bat species (*Rhinolophus* spp.) are widely distributed, including *R. sinicus*, *R. ferrumequinum*, *R. macrotis*, *R. pearsoni*, and *R. pusillus*, and are also the most frequent SARSr-CoV carriers throughout the nation [7,8,20,21,22,27,40,43,45,58,59,61,62,63,68] (Figure 1). The most variable regions among bat SARSr-CoVs are the S and ORF8 genes [62]. The S protein in certain strains is capable of using human ACE2 as a receptor and thus poses a direct threat to humans [69]. Interestingly, all the SARSr-CoVs that are capable of using human ACE2 were found in *R. sinicus* in Yunnan Province [7,22,27,62]. Other SARSr-CoVs that cannot use human ACE2 were distributed in multiple provinces, from north Jilin, Shaanxi, Shanxi to south Hubei, Zhejiang, Yunnan, Guizhou, and Guangdong (Figure 1). Another protein, ORF8, was suggested to be important for interspecies transmission, as most human SARS-CoV epidemic strains contain a signature 29-nucleotide deletion in ORF8 compared to civet SARSr-CoVs, which results in the formation of two separate open reading frames, ORF 8a and 8b [40]. Only two *R. ferrumequinum* and one *R. sinicus* from Yunnan Province carried viruses that possess ORF8 proteins with exceptionally high amino acid identities to that of human/civet SARSr-CoVs [40,62]. It was strongly suggested that SARS-CoV most likely originated from Yunnan *Rhinolophus* bats via recombination events among existing SARSr-CoVs.

These studies revealed that various SARSr-CoVs capable of using human ACE2 are still circulating among bats in China, highlighting the possibly of another SARS-like disease outbreak. Certain areas in Yunnan Province are hotspots for spillover. To support this hypothesis, we provide serological evidence of bat SARSr-CoV infection in humans in Yunnan Province where no prior exposure to SARS-CoV was recorded [70]. The majority of the SARSr-CoVs appear not able to use ACE2, but their infectivity or pathogenesis to humans are still unknown. Frequent interspecies recombination may result in another human infectious coronavirus from these SARSr-CoVs. Furthermore, there are still unanswered questions about SARS, e.g., ‘Why did the first SARS case occur in Guangdong Province, but all the human-ACE2-using SARSr-CoVs were found in Yunnan Province?’ and ’Why does *R. sinicus* in certain areas carry human-ACE2-using SARSr-CoVs but no other *Rhinolophus* species carry the same viruses?’ Above all, further extensive surveillance of SARSr-CoVs in China is warranted.

### 3.2. MERS-Cluster Coronaviruses

Different to bat SARSr-CoV, MERS-cluster CoVs were found in bats before the MERS disease outbreaks. Two bat CoVs, *Tylonycteris* HKU4 and *Pipistrellus* HKU5 were first described as putative group 2c CoVs in 2006 in China. They were associated with the HCoV-EMC (MERS-CoV) that started the 2012 pandemic [9,38,39]. It is generally accepted that Middle East dromedary camels were the major animal source for the zoonotic transmission of human MERS, while bats harbor CoVs that shared common ancestry with MERS-CoV [71]. Extensive global surveys revealed a wide distribution of largely diverged MERS-cluster CoVs (lineage 2c CoVs) [71]. Two closely related *Neoromicia zuluensis* bat CoVs, NeoCoV and PREDICT/PDF-2180, were subsequently found, further supporting the idea that MERS-CoV was descended from an ancestral virus of African bats [72,73]. So far, three species of lineage 2c CoVs have been found in bats, according to the latest CoV taxonomy reports. Based on phylogenetic trees constructed using RdRp, ORF1, S1, and N sequences, bat MERS-related CoVs (MERSr-CoVs) are the closest relatives of MERS-CoV, followed by HKU4-CoV and HKU5-CoV. However, in the S1 region, MERS-CoV was much closer to HKU4-CoV than to MERSr-CoV or HKU5-CoV. Likewise, pseudovirus assays showed that the MERSr-CoV (HKU25 and 422CoV) spike protein can use human DPP4 for entry into hDPP4-expressing cells, although with lower efficiency than that of MERS-CoV or HKU4-CoV spike proteins [49,50]. There is no evidence of HKU5-CoV using the human DPP4 receptor [74].

All three types of bat MERS-cluster CoVs can be found in China (Figure 1 and Figure 2). Their reservoir hosts all belong to the Vespertilionidae family. MERSr-CoV can be found in multiple bat species, including *Pipistrellus* bats (*P. abramus* and *P. pipistrellus*), great evening bats (*Ia io*), particolored bats (*Vespertilio superans*), and Chinese pipistrelle bats (*Hypsugo pulveratus*) [49,50,52]. Due to this wide host spectrum, MERSr-CoV also showed a large genetic diversity, ranging from 72 to 100% in the conserved 440 bp RdRp region. In contrast, HKU4-CoVs were only carried by *Tylonycteris* bats (*T. pachypus* and *T. robustula*) and were relatively conserved [38,39,49] (Figure 2). HKU5-CoVs were found in different *Pipistrellus* bats (*P. abramus*, *P. pipistrellus*, *P. minus*, and *P.* spp.) [8,36,38,39,49,51]. Like HKU4-CoVs, they are also relatively conserved. The range of distribution varies, depending on MERS-cluster CoV species. HKU5-CoVs should be the most widely distributed CoVs among the three as their hosts, *Pipistrellus* bats, live close to humans. However, the reported CoV positive samples can only be found in Guangdong, Hong Kong, and Macau, possibly due to a lack of investigation in other provinces. In contrast, MERSr-CoVs were reported in multiple bat species in Sichuan, Guangdong, and Hong Kong at a much lower level than HKU5-CoVs. Similarly, *Tylonycteris* bats are a rare bat species that live in bamboo, which restricted the distribution of HKU4-CoVs to certain locations in Guangdong, Guangxi, Yunnan, Guizhou, Hong Kong, and Macau (Figure 1). To sum up, it appears that the risk of MERS-cluster CoV spillover to humans leading to an epidemic in China is low for the following reasons: (1) the geographical distribution of MERSr-CoVs and HKU4-CoVs that have the potential to infect humans (capable of using human entry receptors) is limited, and (2) HKU5-CoVs that widely exist in Chinese bats across the nation have not obtained the ability of using human entry receptors. However, we should not underestimate the possibility of recombination among different bat CoVs that lead to the generation of potential pandemic viruses.

### 3.3. HKU2 (SADS)-Related CoV (HKU2r-CoV)

HKU2r-CoVs have only been reported in China and Kenya. From studies in China, HKU2r-CoVs have been frequently found in *Rhinolophus* bats (*R. affinis*, *R. sinicus*, *R. rex*, *and R. pusillus*) in several provinces before the SADS outbreak [2,7,8,38,41,44]. So far, the virus has been reported in Hong Kong, Guangdong, Yunnan, and Tibet. There are perhaps more to be discovered in other provinces considering the wide range of *Rhinolophus* bats. Notably, these bat species, which constantly interact with both livestock and humans in China, also harbor SARSr-CoVs (see Section 3.1). Likewise, HKU2r-CoVs showed a high genetic diversity with SARSr-CoVs (Figure 2). Due to these characteristics, HKU2r-CoVs were listed as viruses that were highly likely to cross species to humans. The novel HKU2r-CoV, swine acute diarrhea syndrome coronavirus (SADS-CoV), was identified as the etiological agent responsible for a large-scale outbreak of fatal disease in pigs in China, Guangdong Province in 2017 [2]. The entry receptor of SADS-CoV has not been identified, yet this virus showed a capacity for infecting a wide range of human, swine, and bat cells (unpublished data). In China, the high density of pig farms and the wide distribution of host bat species promote the possibility of future HKU2r-CoV cross-species transmission [75]. Thus, studies on bat HKU2r-CoVs spillover potential and their pathogenesis are urgent.

## 4. A SADS-CoV Model of Prediction and Other Hotspot Viruses

To predict the next CoV that will cause a virus outbreak in future, we list the general factors that may contribute to this outbreak. Firstly, bats host a large number of highly diverse CoVs. It is known that CoV genomes regularly undergo recombination during infection, and a rich gene pool can facilitate this process. Secondly, bat species are widely distributed and live close to humans. Thirdly, the viruses are pathogenic and transmissible. In this context, SADS-CoV and SARS-CoV outbreaks in China are not unexpected. By this model, there are other CoVs that have not yet caused virus outbreaks but should be monitored.

Within the family Vespertilionidae, the mouse-eared bats (*Myotis*) which favor roosting in abandoned human facilities are also a widespread genus of bats besides *Pipistrellus* bats. They carry a large number and genetically diversified HKU6-CoVs that are closely related to *Myotis ricketti* α-CoV Sax-2011 [36,38]. Moreover, bent-winged bats (*Miniopterus* spp.) carry a large variety of α-CoVs. One of the most frequently detected viruses is HKU8-CoV, which was first described circulating in *M. pusillus* in Hong Kong in 2005. Later, it was also found in *M. magnate*, *M. fuliginosus*, and *M. schreibersii* in Hong Kong, Guangdong, Yunnan, Fujian, and Hubei provinces, showing a great genetic diversity [32,33,34,35,37,41,60] (Figure 1). Besides HKU8-CoVs, bent-winged bats (*Miniopterus* spp.) also harbor a large amount of *Miniopterus* bat CoV 1 (BtMiCoV-1), which were called CoV1A or CoV1B previously. This viral species was found almost as frequently as HKU8-CoV in multiple provinces in China in *Miniopterus* bats, although these viruses showed a relatively small sequence variation between each other [32,33,34,35,37,41,60]. Genetic analysis indicates that BtMiCoV-1, HKU8-CoV, and HKU7-CoV (previous name) are different but closely related CoVs circulating in bent-winged bats and may have descended from a common ancestor [34]. Additionally, *Rousettus leschenaultii* bats in the family of Pteropodidae harbor HKU9-CoVs. As a fruit bat, *Rousettus leschenaultii* has a wider flying range than most of the insectivorous bats in China, thus it may carry viruses over long distances. A comparison of the reported HKU9-CoV sequences showed a high genetic diversity within this viral species [55,56,57] (Figure 2). The last CoV that should be mentioned is HKU10-CoV. HKU10-CoVs can be found in bats from different genera (*Rousettus leschenaultii* and *Hipposideros pomona*), suggesting interspecies transmission between bats [7,26,27,39]. A genetic difference can also be observed for this virus species (Figure 2). Above all, these viruses fit well in our SADS prediction model and should be monitored in our future studies.

## 5. Other Bat CoVs in China

In 2016, a novel β-CoV, Ro-BatCoVGCCDC1, was identified from the *Rousettus leschenaultii* bat. However, we confirmed the host was a closely related *Eonycteris spelaea* bat upon species identification and then renamed the virus as BtEoCoV-GCCDC1 (Table 1). The uniqueness of this virus is that it contains a gene that most likely originated from the p10 gene of a bat orthoreovirus [53]. A two-year follow-up study also illustrated that BtEoCoV-GCCDC1 persistently circulates among bats. Different to the genetically diverged HKU9-CoV, this virus is highly conserved (Figure 2). BtEoCoV-GCCDC1 has only been found in south Yunnan Province so far [54,55]. In addition, there are other bat CoVs that have been identified in China: *Rhinolophus ferrumequinum* α-CoV HuB-2013 [8], *Myotis ricketti* α-CoV Sax-2011 [8,37], *Nyctalus velutinus* α-CoV SC-2013 [8], *Scotophilus* bat CoV 512 [37], *Hipposideros* bat β-CoV Zhejiang2013, and a *Murina leucogaster* bat CoV, which has been described as the evolutionary ancestor of PEDV [37]. Notably, there are still many unclassified bat CoVs circulating in China, particularly in the northern part of the nation where bat viruses were rarely studied (Figure 1). According to the criteria defined by the ICTV, the CoV family will most likely expand following further investigation of bat CoVs in China.

## 6. Coexistence of Different Coronaviruses or Other Viruses in Bats

The coexistence of more than two viruses in the same bat is quite common for some bat species. The coexistence of *Miniopterus* bat CoV 1 and HKU8-CoV in one bat has been frequently reported [7,34]. Another example is the coexistence between *Rhinolophus* HKU2-CoVs (SADS-CoV) and SARSr-CoVs that caused the virus outbreaks, respectively [2,45]. Real-time monitoring this bat genus is necessary for the prevention of future SARS-like outbreaks. Moreover, two or more distinct genotypes of HKU9-CoVs were reported to coexist in a single *Rousettus* bat [56]. The coexistence of HKU9-CoVs and a new identified bat filovirus (Mengla virus) that is phylogenetically related to the Ebola and Marburg viruses was also identified from *Rousettus* bats [17,55]. Given that a bat orthoreovirus p10 gene was incorporated in the BtEoCoV-GCCDC1 genome, recombination between the bat filovirus and HKU9-CoV cannot be excluded. Other pairs were also recorded—HKU8-CoV with unclassified α-CoV [7], HKU2-CoV with unclassified α-CoV [7], HKU10-CoV with unclassified β-CoV [7], and HKU6-CoV with bat adenovirus [36].

## 7. Conclusions

Two bat origin CoVs caused large-scale epidemics in China over fourteen years, highlighting the risk of a future bat CoV outbreak in this nation. In this review, we have summarized the current findings related to bat CoV epidemiology in China, aiming to explore the associations between CoV species, bat species, and geographical locations, and eventually we aim to predict the cross-species transmission potential of these bat CoVs. Admittedly, the analysis may be affected by inaccurate or incomplete data. For example, not all research groups performed bat species identification or used Global Positioning System (GPS) during bat sampling. Bats in the north or west provinces were not surveyed either. Nonetheless, we believe this analysis is a good starting point for further research. Moreover, there are other outstanding questions that should be addressed in future studies: (1) given that most of the ICTV classified CoV species are from bats, why there are so many genetically divergent CoVs in bats, (2) the pathogenesis of most bat CoVs in humans remains unknown as the viruses have never been isolated or rescued—apart from the viruses identified during the outbreaks, many viruses pose a threat to human health, (3) although SARS-CoV and SADS-CoV were known to be transmitted from bats to human or swine, their exact transmission routes are unknown, and (4) why bats can maintain CoVs long-term without showing clinical symptoms of diseases. A unique bat immunity model has been proposed. The authors have shown that constitutively expressed bat interferon α may protect bats from infection [76], while some particularly dampened immune pathways may allow bats to have a higher tolerance against viral diseases [77]. While we start to unveil the mystery of unique bat immunity, there is still long way to go before we can fully understand the relationship between bats and coronaviruses.

## Figures and Tables

**Table 1:** International Committee of Taxonomy of Viruses (ICTV) classification of coronaviruses species, reservoir hosts, and presence reported in China.

Coronavirus Species	Abbreviations	Human	Bats	Other Animals	Reported in China	
Bat coronavirus HKU10	BtCoV-HKU10		Yes		Yes [7,8,26,27]	**α-CoV**
Bat coronavirus CDPHE15	BtCoV-CDPHE15		Yes		No	
*Rhinolophus ferrumequinum* alphacoronavirus HuB-2013	BtRfCoV-HuB13		Yes		Yes [8]	
* Human coronavirus 229E	HCoV-229E	Yes			Yes [28,29]	
Lucheng Rn rat coronavirus	LRNV			Yes (rat)	Yes [30]	
Ferret coronavirus	FRCoV			Yes (ferret)	No [31]	
* Mink coronavirus 1	MCoV			Yes (mink)	No [14]	
*Miniopterus* bat coronavirus 1	BtMiCoV-1		Yes		Yes [7,8,32,33,34,35,36,37]	
*Miniopterus* bat coronavirus HKU8	BtMiCoV-HKU8		Yes		Yes [7,8,33,34,35,37,38,39,40,41]	
*Myotis ricketti* alphacoronavirus Sax-2011	BtMy-Sax11		Yes		Yes [8,37]	
*Nyctalus velutinus* alphacoronavirus SC-2013	BtNy-Sc13		Yes		Yes [8]	
* Porcine epidemic diarrhea virus	PEDV			Yes (pig)	Yes [42]	
*Scotophilus* bat coronavirus 512	BtScCoV-512		Yes		Yes [37]	
* *Rhinolophus* bat coronavirus HKU2 (SADS)	BtRhCoV-HKU2		Yes	Yes	Yes [2,7,8,38,43,44,45]	
* Human coronavirus NL63	HCoV-NL63	Yes			Yes [28,29]	
NL63-related bat coronavirus strain BtKYNL63-9b	BtKYNL63		Yes		No [24]	
* Alphacoronavirus 1 (Transmissible gastroenteritis virus)	TGEV			Yes (pig)	Yes [42]	
China *Rattus* coronavirus HKU24	RtCoV-HKU24			Yes (rat)	Yes [46]	**β-CoV**
* Human coronavirus HKU1	HCoV-HKU1	Yes			Yes [28,29]	
* Murine coronavirus (Murine hepatitis coronavirus)	MHV			Yes (mouse)	No [47]	
Bat *Hp*-betacoronavirus Zhejiang2013	BtHpCoV-ZJ13		Yes		Yes [8]	
Hedgehog coronavirus 1	EriCoV-1			Yes (hedgehog)	No [48]	
* Middle East respiratory syndrome-related coronavirus	MERSr-CoV	Yes	Yes		Yes [49,50]	
*Pipistrellus* bat coronavirus HKU5	BtPiCoV-HKU5		Yes		Yes [38,39,49,51,52]	
*Tylonycteris* bat coronavirus HKU4	BtTyCoV-HKU4		Yes		Yes [36,38,39,49,50,51]	
*Rousettus* bat coronavirus GCCDC1	^#^ BtEoCoV-GCCDC1		Yes		Yes [53,54,55]	
*Rousettus* bat coronavirus HKU9	BtRoCoV-HKU9		Yes		Yes [39,55,56,57]	
* Severe acute respiratory syndrome-related coronavirus	SARSr-CoV	Yes	Yes		Yes [7,8,20,21,22,27,37,40,45,58,59,60,61,62,63,64]	
* Betacoronavirus 1 (Human coronavirus OC43)	HCoV-OC43	Yes			Yes [28,29]	
Wigeon coronavirus HKU20	WiCoV-HKU20			Yes (bird)	Yes [65]	**δ-CoV**
Bulbul coronavirus HKU11	BuCoV-HKU11			Yes (bird)	Yes [65]	
Coronavirus HKU15	PoCoV-HKU15			Yes (pig)	Yes [66]	
Munia coronavirus HKU13	MuCoV-HKU13			Yes (bird)	Yes [65]	
White-eye coronavirus HKU16	WECoV-HKU13			Yes (bird)	Yes [65]	
Night heron coronavirus HKU19	NHCoV-HKU19			Yes (bird)	Yes [65]	
Common moorhen coronavirus HKU21	CMCoV-HKU21			Yes (bird)	Yes [65]	
*^?^ Beluga whale coronavirus SW1	BWCoV-SW1			Yes (whale)	No [13]	**γ-CoV**
* Avian infectious bronchitis virus	IBV			Yes (bird)	Yes [12]	

* The disease-causing CoVs are indicated and the three zoonotic CoVs are in bold. *? BWCoV-SW1 was found in a sick whale, but whether it was the etiological agent of the infection was not proven. # Carrier of this virus was confirmed as *Eonycteris spelaea*, but not *Rousettus* bats. The virus was renamed accordingly.
